# Closed medial total subtalar joint dislocation without ankle fracture: a case report

**DOI:** 10.1186/1752-1947-8-313

**Published:** 2014-09-20

**Authors:** Mohamed Azarkane, Hassan Boussakri, Abdelghani Alayyoubi, Mohamed Bachiri, Abdelhalim Elibrahimi, Abdelemejid Elmrini

**Affiliations:** 1 Department of Orthopedic Surgery and Traumatology, B4 UHC Hassan II, Fes, Morocco

**Keywords:** Ankle, Dislocation, Subtalar joint

## Abstract

**Introduction:**

Total subtalar dislocation without fracture of the ankle is a rare clinical entity; it is usually due to a traumatic high-energy mechanism. Standard treatment is successful closed reduction under general anesthesia followed by non-weight bearing and ankle immobilization with a below-knee cast for 6 weeks.

**Case presentation:**

We present the case of a 30-year-old Moroccan woman who was involved in a road traffic accident. She subsequently received a radiological assessment that objectified a total subtalar dislocation without fracture of her ankle. She was immediately admitted to the operating theater where an immediate reduction was performed under sedation, and immobilization in a plaster boot was adopted for 8 weeks. The management of this traumatic lesion is discussed in the light of the literature.

**Conclusions:**

Medial subtalar dislocation is a rare dislocation and is not commonly seen as a sports injury because it requires transfer of a large amount of kinetic energy. The weaker talocalcaneal and talonavicular ligaments often bear the brunt of the energy and are more commonly disrupted, compared to the relatively stronger calcaneonavicular ligament. Urgent reduction is important, and closed reduction under general anesthesia is usually successful, often facilitated by keeping the knee in flexion to relax the gastrocnemius muscle. Long-term sequelae include talar avascular necrosis and osteochondral fracture, as well as chronic instability and pain.

## Introduction

According to Broca [1] “It is a dislocation in which the talus maintains its relationship with the bones of the leg, while the calcaneus and the navicular move under it”. A total subtalar joint dislocation was first described in 1811 by Judcy [2]. This is a rare disease that is becoming increasingly common. It constitutes 15% of injuries on the talus, and 1 to 2% of all dislocations [3]. It mainly affects young men following inversion trauma of a foot in equinus. This case report describes a medial subtalar joint dislocation in a patient who had been involved in a road traffic accident.

## Case presentation

**Figure 1:**
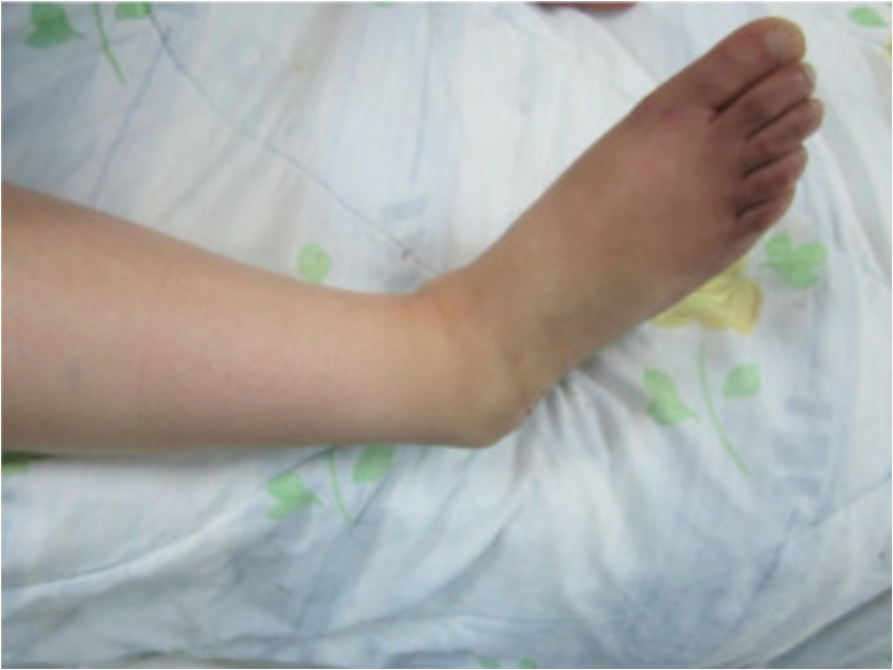
Before reduction, showing inversion of the foot.

**Figure 2:**
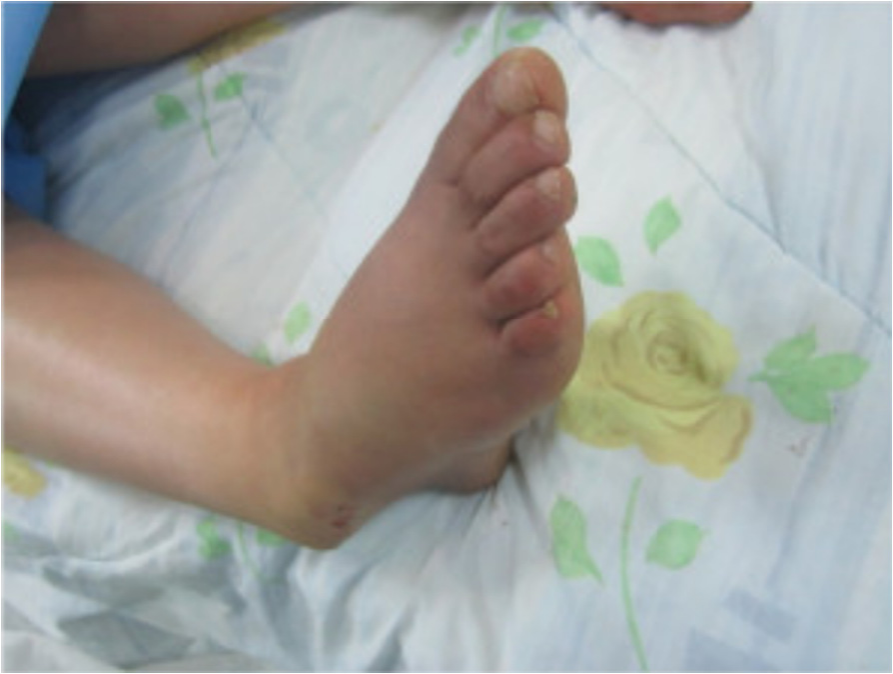
Before reduction, prominent head of talus.

**Figure 3:**
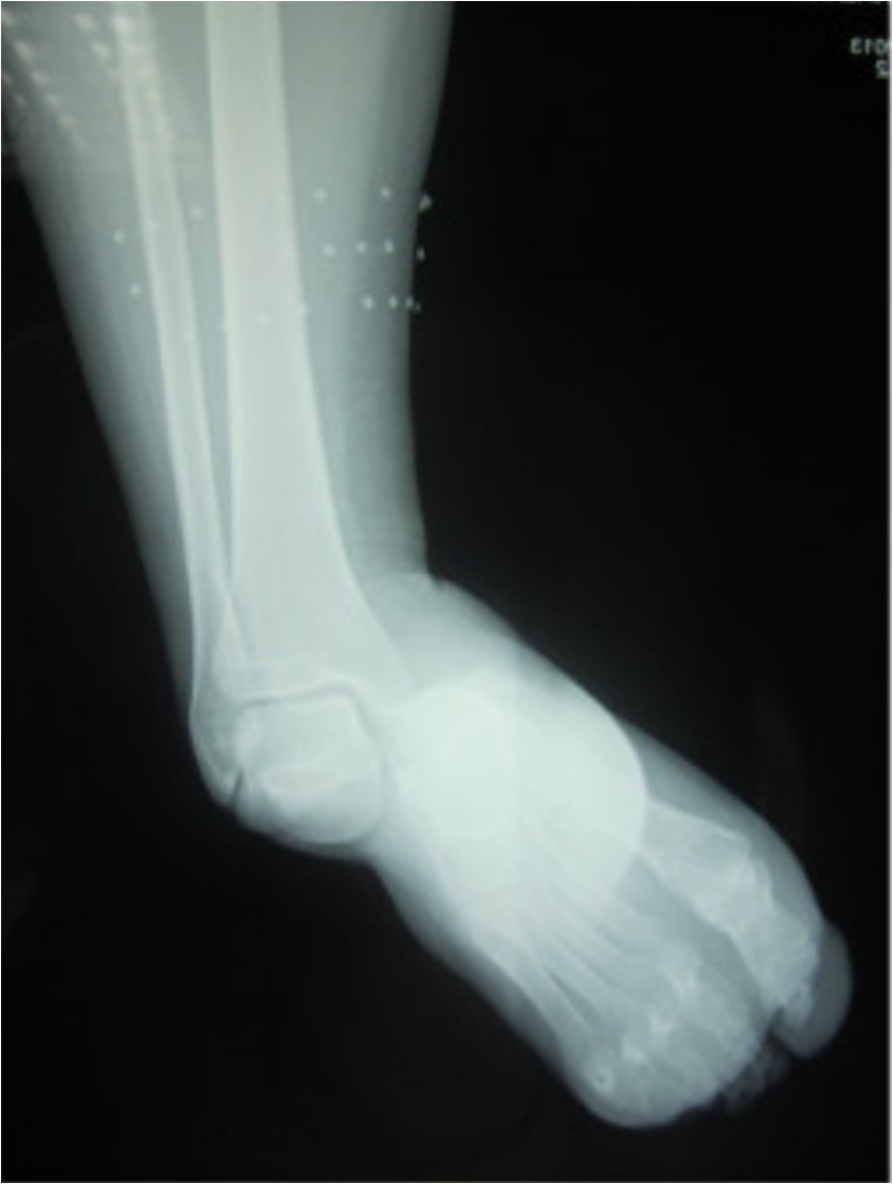
Anteroposterior radiograph of the ankle showing medial subtalar dislocation without fractures.

**Figure 4:**
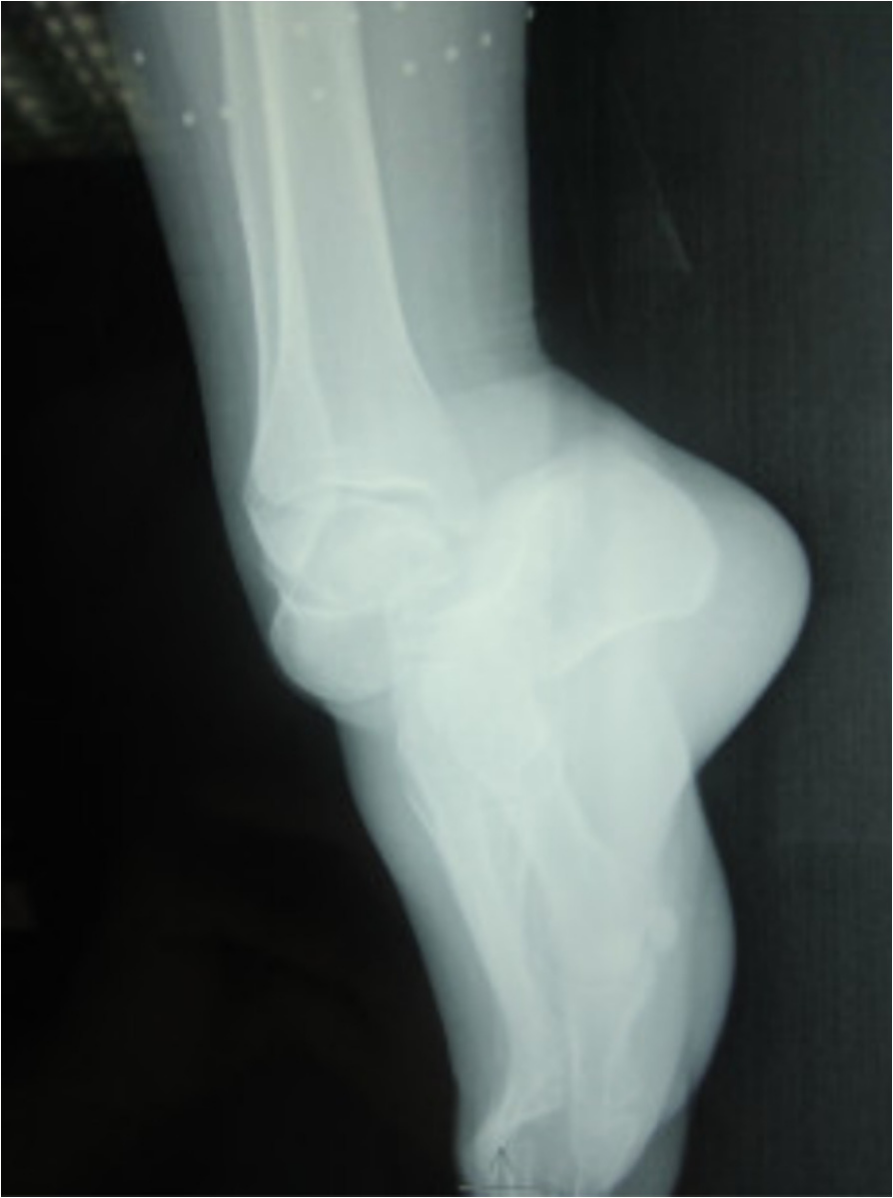
Lateral radiograph of the ankle showing medial subtalar dislocation without fractures.

**Figure 5:**
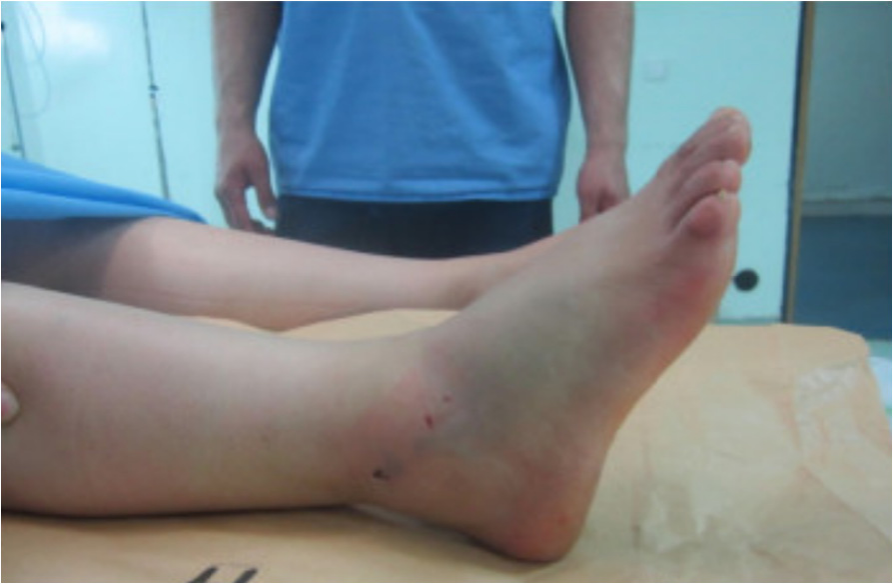
Clinical aspect after reduction lateral view.

**Figure 6:**
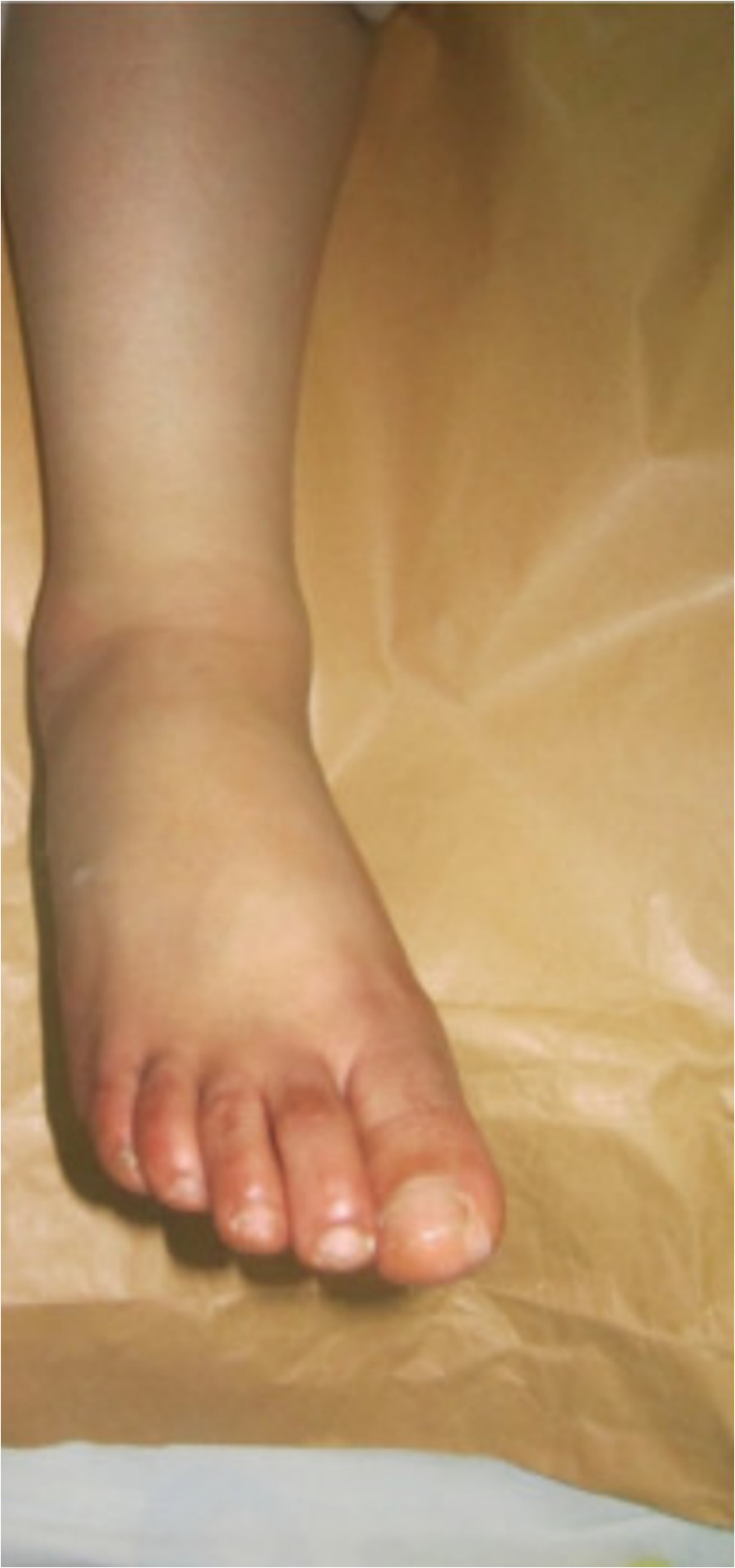
Clinical aspect after reduction anterior view.

**Figure 7:**
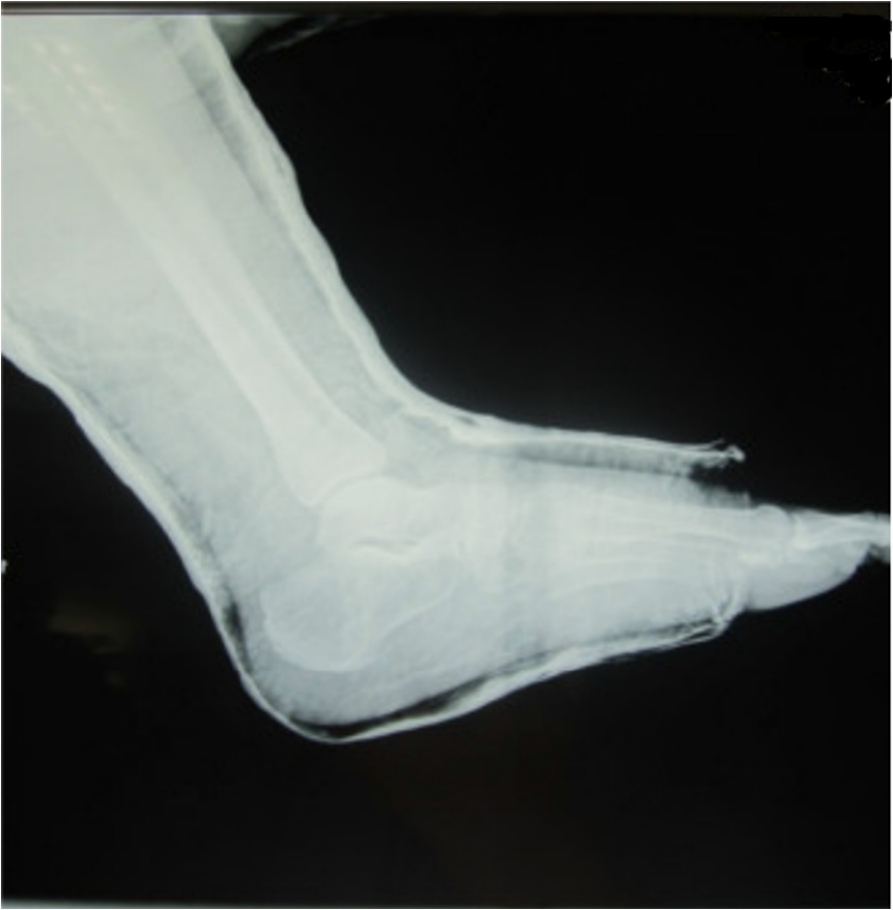
Control lateral radiographs after reduction.

**Figure 8:**
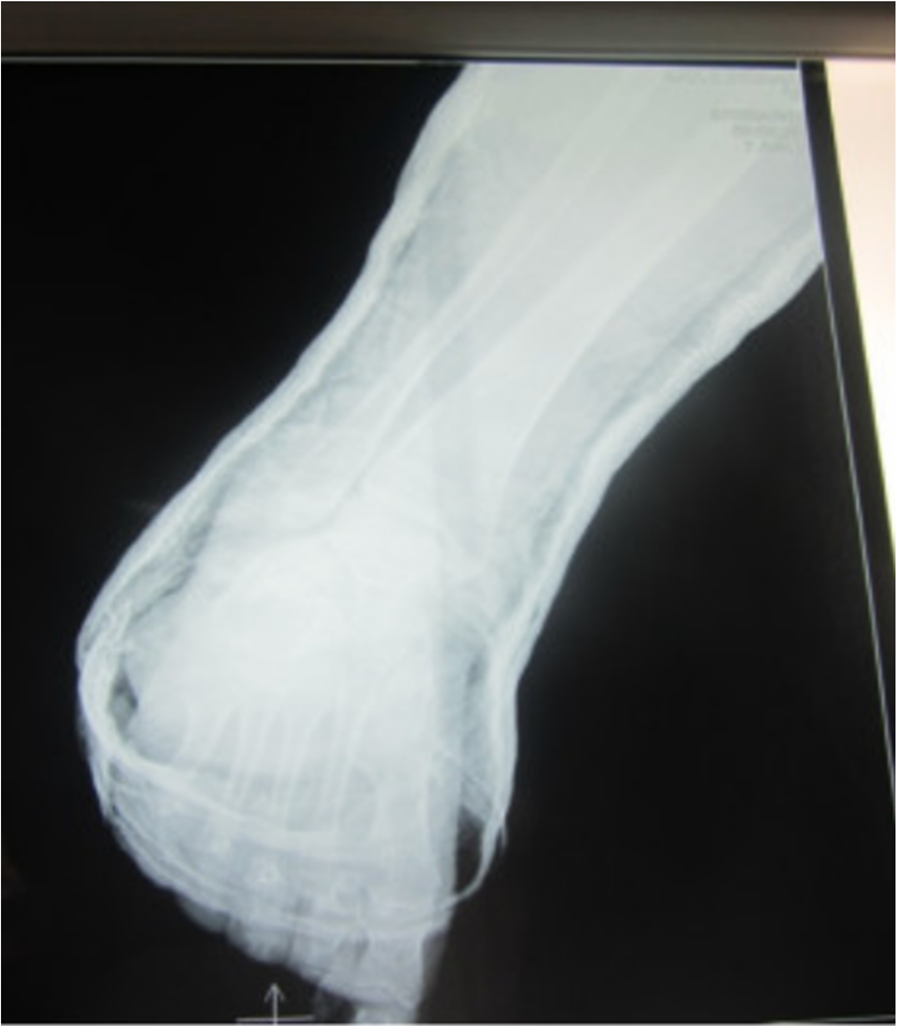
Control anteroposterior radiographs after reduction.

A 30-year-old Moroccan woman, with no significant medical history, was involved in road accident (she was a pedestrian struck by a car) with the point of impact on her right foot. The trauma mechanism was not precise. She was admitted 1 hour after the trauma to our emergency unit with pain and total functional impairment of her right lower limb. Clinical examination noted a deformation of her right foot: her heel had been displaced inwards in relation to her leg, her foot being in inversion, plantar flexion and adduction, with shortening of the medial border of her foot (“acquired clubfoot”), and the skin had been stretched over the protrusion of talus without any wound. The head of talus was palpable at the dorsolateral aspect of her ankle below the lateral malleolus (Figures 1 and 2). Any active or passive mobilization of her foot was impossible. She subsequently received a radiological assessment that objectified a total loss of contact between her talus and her calcaneus, and a shift of her calcaneus and the navicular bone block internally while her talus maintained its contact with her tibia performing medial subtalar dislocation without associated fracture (Figures 3 and 4). She was immediately admitted to the operating theater where an immediate reduction was performed under sedation. With her knee in flexed position to relax her Achilles tendon, the reduction was successfully performed by an external maneuver; traction with direct pressure on the projection of her talus and eversion of her foot (Figures 5 and 6). Reduction was achieved easily, we found a good stability after reduction, and immobilization in a plaster boot was adopted for 8 weeks (Figures 7 and 8). Removal of plaster was performed after 8 weeks. We started rehabilitation to enable the proprioceptive response, muscle building, and to recover the range of motion of her ankle and subtalar joint. She was monitored regularly in our out-patient clinic with a follow-up of 12 months. We evaluated the functional results of our patient according to Gay and Evrard’s criteria based on a study of pain, walking, mobility, stability and professional activities. We noted good functional results with 3 points for each criterion adding up to 15 points.

## Discussion

Subtalar dislocations are rare but not exceptional. Leitner [3] estimated that these dislocations represent 1% of all dislocations, and DeLee and Curtis [4] reported that they represent a little less than 2% of dislocations of all major joints, these two expressions of the frequency of subtalar dislocations are the most cited in the literature. This is a condition that mainly affects young men following a high-energy trauma. The mechanism of injury is due to an inversion of the forefoot blocked in equinus. This condition is frequently encountered in sports injuries involving landing from jumps (basketball, volleyball, dance and so on …). Dislocation can be medial, lateral, anterior or posterior. The medial variety is the most common. The greater frequency of internal displacements can be simply explained by the fact that the subtalar joint is actually unstable in inversion [5,6]. The mechanism for medial subtalar dislocation is forceful inversion of the foot blocked on the ground causing ligament tears in a specific chronological manner: firstly, the dorsal talonavicular ligament is injured, then the two interosseous ligaments and finally the calcaneofibular ligament. This dislocation usually occurs after a high-energy trauma, rarely a trivial inversion after a sports injury. Jarde *et al*. [5] reported that the diagnosis of subtalar dislocation is clear due to the foot deformity, which can be masked by significant edema sometimes. A front and profile ankle X-ray confirms the dislocation by showing the talus bone in place in the tibiofibular mortise while the foot is warped inward. This dislocation may be associated with skin injuries; Merchan [7] found 41% of cases of cutaneous opening in a series of 39 cases. It may also be accompanied by a fracture: intra-articular fractures which are osteochondral fractures of the articular surfaces beneath the talus or astragalo-scaphoid. In this situation the prognosis is complicated by the risk of arthrosis they cause. They most often require surgical reduction. Or extra-articular fractures: fractures adjacent to the subtalar joint.

These extra or intra-articulars fractures also influence the prognosis due to the prolonged immobilization they require for their consolidation and can facilitate stiffness and osteoporosis [4]. Reduction must be done urgently, by traction of the foot with the knee flexed to relax the triceps surae muscle. An irreducibility may be the result of interposition of the fibular muscle tendons, ligament, the extensor digitorum brevis muscle or a bone fragment for medial dislocation, but the reduction is often easy under premedication or general anesthesia. Surgery is rarely necessary. The reduction is usually stable and does not require synthesis. The knee should be flexed to relax the Achilles tendon and the foot should be equinus before ridging the calcaneonavicular pedal [8]. The reduction must be maintained in a boot cast for 6 to 8 weeks without support. The evolution shows that the necrosis of the talus is rare [9,10]. Long-term prognosis is good except in cases of cutaneous opening or associated fracture that may cause subtalar arthrosis.

## Conclusions

Subtalar dislocations are rare but serious injuries of the talus. Medial subtalar dislocation is the most common variety. Young sports men are the most affected; this injury is usually due to high-energy trauma. Early diagnosis and urgent reduction are the prerequisites for a satisfactory functional outcome. The prognosis is generally good, although long-term monitoring is required to combat the appearance of subtalar arthrosis.

## Consent

Written informed consent was obtained from the patient for publication of this case report and any accompanying images. A copy of the written consent is available for review by the Editor-in-Chief of this journal.

## Competing interests

The authors declare that they have no competing interests.

## Authors’ contributions

AM drafted the manuscript. BH helped to draft the manuscript. AA helped to draft the manuscript. BM helped to draft the manuscript. E Abdelhalim conceived of the study, and participated in its design and coordination and helped to draft the manuscript. E Abdelemejid conceived of the study, and participated in its design and coordination and helped to draft the manuscript. All authors read and approved the final manuscript.
